# Measurement Invariance of the Prosocial Behavior Scale in Three Hispanic Countries (Argentina, Spain, and Peru)

**DOI:** 10.3389/fpsyg.2020.00029

**Published:** 2020-01-28

**Authors:** Manuel Martí-Vilar, César Merino-Soto, Lucas Marcelo Rodriguez

**Affiliations:** ^1^Departament de Psicologia Bàsica, Facultat de Psicologia, Universitat de València, Valencia, Spain; ^2^Instituto de Investigación de Psicología, Universidad de San Martín de Porres, Lima, Peru; ^3^Centre for Interdisciplinary Research in Values, Integration and Social Development, Pontifical Catholic University of Argentina, Buenos Aires, Argentina

**Keywords:** prosocial, measurement invariance, social behavior, intercultural, university students, validation, assessment

## Abstract

In a growing context of multiculturalism, prosocial behavior is important to build effective social exchange and service orientation among university students. The present study investigates prosocial behavior from a psychometric approach, to obtain evidence of the internal structure of the prosocial behavior scale (PS), in 737 young people enrolled at universities in Argentina (207), Spain (310), and Peru (220). First, the clarity of the items was explored in the three countries; second, possible irrelevant patterns of response, such as the careless and extreme responses, were evaluated; third, the non-parametric Mokken methodology was applied to identify the basic properties of the scale score; fourth, the structural equation modeling (SEM) methodology was used to identify the properties of the internal structure (dimensionality, tau-equivalence) of the latent construct; fifth, the measurement invariance according to sex (intra-equivalence) and country (inter-equivalence) was examined with the SEM methodology and other complementary strategies. Finally, reliability and internal consistency were evaluated both at score level and at item level. Implications for use of the PS instrument are discussed.

## Introduction

Prosocial behavior includes those actions tending to help or benefit other people, irrespective of the intention to be pursued with this help. Such behavior is the result of multiple individual and situational factors including parental variables and empathic traits (Eisenberg and Fabes, 1998). It is understood as a tendency to give rise to actions, belonging to the sphere of habits, practices and social interactions, that are characterized by the beneficent effects they produce on another person (Caprara, 2005). Moreover, Roche (2010) argued that truly prosocial behavior consists of help given to other people or groups in the absence of extrinsic or material reward. There are several different types of actions that make up prosocial behavior, such as physical and verbal help, material giving, verbal comfort, confirmation and positive appreciation of the other, deep listening, empathy, and solidarity, as well as the expression of unity with others (Roche, 1999).

Research on prosociality in diverse cultures has increased over the last few decades (Murakami et al., 2016; Luengo et al., 2017; Rodriguez et al., 2017; Gerbino et al., 2018). This has allowed researchers to carry out several meta-analysis studies on prosociality (Malti and Krettenauer, 2013; Shariff et al., 2016; Mesurado et al., 2019b), that show the value of clinical and educational interventions in encouraging prosocial behavior. For example, based on their own meta-analysis, Mesurado et al. (2019b) concluded that intervention programs aimed at promoting prosocial behaviors showed moderate effectiveness, while intervention programs focused on the prevention of aggressive were highly effective.

Since the construct of prosociality implicates a wide range of different behaviors, its measurement distinguishes between indicators of global prosocial behavior and prosocial behavior expressed in specific situations (Carlo and Randall, 2002). Measures of global prosocial behavior are defined as measures that evaluate personal tendencies to exhibit a series of prosocial behaviors across diverse social contexts and for different motives. An example of this type of global measure is the Prosociality Scale of Caprara et al. (2005). These global measures tend to characterize certain people as prosocial, distinguishing them from others who are not. However, global measures have limited application in research, since they do not investigate possible moderators such as in-group and out-group effects on tendencies to help, among other contextual factors. In contrast, measures of prosocial behavior in specific situations can provide information about more tightly delimited conceptualizations of prosociality, as well as supporting the elaboration and intercorrelation of different types of prosocial behavior. One example of this point is research that distinguishes between different recipients of aid, in terms of measuring the prosociality directed toward relatives, friends and strangers in adolescent populations (Padilla-Walker and Christensen, 2011; Padilla-Walker et al., 2015; Mesurado et al., 2019a). Such specific measures see prosociality as a multidimensional construct, which can be a very beneficial approach when studying interactions between prosociality and other variables (Carlo and Randall, 2002). However, the usefulness of a global or specific approach to measuring prosociality is not intrinsic to the measure itself, but is conditioned by the purpose of its use in basic or applied research, or in professional practice.

Another example of global prosociality measures is the Prosociality Scale (PS; Caprara et al., 2005), which describes the individual variability of prosocial behavior as a stable attribute, and is designed for young adults. It consists of 16 items to answer on an ordinal scale of 5 options ranging from “never/almost never” to “always/almost always.” Based on the original study by the instrument’s authors (Caprara et al., 2005), we can distinguish psychometrically the items that provide high information (items 3, 5, 7, 8, 10, 12, and 13), moderate information (items 4, 6, and 9) and low information (items 1, 2, 11, 14, 15, and 16). The PS has had some international diffusion, with studies in various countries. For example, investigations have been conducted with Colombian adolescents using the reduced version of the scale (Luengo et al., 2017). Studies have also been carried out in Japan (Murakami et al., 2016), and in a sample of Argentinian adolescents (Rodriguez et al., 2017). In the latter study a confirmatory factor analysis arrived at a scale of two dimensions (*prosocial behavior* and *empathy and emotional support*) while reducing the number of items to 10, and achieving an internal consistency of α = 0.78. Cross-cultural work has also been carried out on samples of children from Colombia, Italy, Jordan, Kenya, the Philippines, Sweden, Thailand, and the United States (Pastorelli et al., 2016), although data on the reliability and validity of the Prosociality Scale instrument were not presented in that study. It is worth mentioning that the aforementioned studies were carried out on children and adolescents, an age range for which the scale of Caprara et al. (2005) was not specifically designed. Their results should therefore be interpreted with caution, and should not automatically be generalized to adult populations.

Since the Prosociality Scale is recognized internationally, it is of great scientific and practical interest to evaluate its psychometric characteristics and variance across diverse populations. Additionally, studies that use a version of the scale in Spanish are particularly valuable since they are moderately scarce compared to studies that a use a version in English. Indeed, a recent systematic review of measures of prosocial behavior (Martí-Vilar et al., 2019) reported that PS is among the measures with few validation studies carried out adults, but with excellent internal consistency. The relationship between the importance of the construct and the its measurement in adults does not seem to be isomorphic, since there are few validation studies of internal structure and correlation studies with other relevant constructs: except for a study by Rodriguez et al. (2017), this information is practically absent in the Ibero-American population. These authors performed a confirmatory factor analysis on a population of Argentinian adolescents. In their study, a 10-item model with two dimensions was obtained, namely prosocial behavior on the one hand and empathy and emotional support on the other. In turn, they analyzed the convergent validity of the instrument, obtaining significant correlations with some dimensions of the scale of prosocial tendencies produced by Carlo and Randall (2002).

Investigations that have used the Prosociality Scale have rarely addressed certain aspects that could help to understand its psychometric functioning. For example, the functioning of the items within a tau-equivalent model has not been analyzed; this property is a condition for the use of the reliability coefficient type (Graham, 2006; Trizano-Hermosilla and Alvarado, 2016), as well as for identifying the homogeneity of the representation of the content and interpretation of the score. In this sense, because the factor loads signify the strength with which the items are connected to (represent) the latent construct (Trizano-Hermosilla and Alvarado, 2016), the similarity or dissimilarity of factor loads can influence interpretation of the score. Therefore, different factor load patterns (e.g., item 1: 0.80, item 2: 0.50, item 3: 30, item 4: 0.30; compared with item 1: 30, item 2: 30, item 3: 50, item 4:0.80), may not lead to the same interpretation of the construct.

On the other hand, all studies that have used the Prosociality Scale (except Caprara et al., 2005) have applied linear models that included latent variables (in other words, structural equation modeling, or SEM); however, a deeper analysis of the instrument requires considering that the interpretation rests on the score observed, and therefore a non-parametric methodology that uses the observed score as the main reference for the adjustment of the items may be necessary, and a prerequisite for the application of parametric models such as linear SEM modeling (Dima, 2018). The sequential or joint application of several procedures to identify the psychometric properties of a measure can be better understood within a framework of sensitivity analysis, in which the results of various methods or modifications of the data are contrasted, in order to evaluate the eventual convergence. This has been especially applied in the investigation of equivalence of measures (Hambleton, 2006; Teresi et al., 2009) and adaptation of evidence (Dima, 2018). Finally, due to the different informative strength of each PS item (as found by Caprara et al., 2005), it is plausible that each item is differently sensitive to factors such as sex; in this sense, the differences between groups in the means can mask fine differences at the item level. More precisely, descriptive analysis at the item level is relevant because each unit represents an elementary behavior of the intended construct, and its statistical behavior can help to better understand this, and precede the use of advanced analyses (Dima, 2018). Additionally, due to the apparent tendency to use single-item scales in self-report and epidemiological investigations, information at the item level can contribute to more informed choices in such uses.

The aim of the present study was to evaluate the psychometric functioning of the Prosocial Conduct Questionnaire in a context of intercultural use, focused on university participants from three Spanish-speaking countries: Argentina, Spain, and Peru. Specifically, the central objective was to obtain evidence of the validity of the internal structure of the Prosocial Behavior Questionnaire in three Hispanic countries, through the exploration of scalability, dimensionality, invariance of measurement and reliability of internal consistency. The aspects evaluated in this study may be specific to their use in these countries, and are linked to the evidence on the internal structure of the scale, which is a key component for other sources of evidence of validity (Lewis, 2017). Dimensionality, invariance and reliability can be considered fundamental contributors to the valid interpretation of a score, and together define an instrument’s internal structure (Rios and Wells, 2014); that is, the theoretically coherent relationship between the components of a measure that serve as a basis for the interpretation of the score (American Educational Research Association [AERA] et al., 2014). Accordingly, evidence of validity based on the internal structure is critical in conditioning other evidence of validity (Ziegler and Hagemann, 2015). In the present study, scalability was also evaluated as a property of the score for establishing ordinal differences between subjects based on their observed scores (Mokken, 1971; van Schuur, 2003; Smits et al., 2012). This aspect is not necessarily equal to the dimensionality of an instrument, and therefore must be evaluated in a complementary way (Smits et al., 2012), usually with the non-parametric approach of Mokken (1971). The equivalence or invariance of measurement, as well as the similarity of internal consistency, and the sex differences in the level of total score and individual item, were also considered. Apparently, this is the first study that tests the dimensionality and invariance of the Prosociality Scale in several Ibero-American countries, and thus represents an advance toward the global use of the instrument.

## Materials and Methods

### Participants

The study population were adult university students of Psychology, residing in Spanish-speaking metropolitan cities. The collected sample comprised 737 subjects, from Spain (*n* = 310), Peru (*n* = 220), and Argentina (*n* = 207), 568 being female (77.2%, the rest were all male). The distribution of sexes across the three countries (Argentina: 176 women, 85.0%, Peru: 143 women, 65.3%; Spain: 249 women, 80.3%) was moderately similar (Shanon index, *H*_*male*_ = 0.451, *H*_*female*_ = 0.465). Although there were statistically significant differences in the sex distributions (Marascuilo and McSweeney method, Marascuilo and McSweeney, 1967) between Peru and Argentina on the one hand, and Spain and Argentina on the other, these were moderate (*d* = 0.63) and small (*d* = 0.45), respectively; and overall they were small (Cohen-*w*_*adjusted*_ = 0.273, Sheskin, 2007). The academic semesters sampled were the first (138, 18.8%), second (105, 14.3%), third (188, 25.5%), fourth (208, 28.3%), and fifth (97, 13.2%) semesters.

The total age in the sample was: M = 21.42, *SD* = 4.11, Min = 16, Max = 53); between the samples (Argentina: *M* = 20.67, *SD* = 2.88; Spain: *M* = 21.66, *SD* = 4.35; Peru: *M* = 21.79, *SD* = 4.66), the differences were statistically significant (*F*[2,733] = 4.926, *p* < 0.01) but the effect size (ω*^2^* = 0.01) was very small (Field, 2013). The differences between distribution of semesters in Peru and Spain (Kolmogorov–Smirnov *D* = 0.386, *p* < 0.01), and Peru and Argentina (Kolmogorov–Smirnov *D* = 0.433, *p* < 0.01) were statistically significant, while those for Spain and Argentina were not (Kolmogorov–Smirnov: *D* = 0.084, *p* > 0.10). But the practical significance of these differences, in terms of similarity of frequencies (overlap, PSR, Rom and Hwang, 1996) tended to be high: PSR Peru–Spain = 80.7%; PSR Peru–Argentina = 78.4%; PSR Spain Argentina = 95.8%. According to previous studies of the validation and substantive use of the instrument in the adult population (Murakami et al., 2016; Pastorelli et al., 2016; Luengo et al., 2017; Rodriguez et al., 2017), the various sub-samples of our participants were not differentiated from one another in relation to sampling (non-probabilistic), coverage (young adults), or main activity (university studies), and therefore they can be thought of as generally aligned.

### Instruments

#### Demographic Sheet

A questionnaire was compiled to gather sociodemographic information, namely country, city, age, sex, level of studies, and academic semester.

#### Prosociality Scale (Caprara et al., 2005)

This is a self-report measure that quantifies prosociality as a stable attribute in the adult population. It consists of 16 ordinally scaled items each with five response options. The response instructions posit a generic and timeless context of prosocial behaviors. In relation to the internal consistency of the instrument, the original authors reported unidimensionality, a wide range of psychometric precision, internal validity of the items, and internal consistency of α = 0.91 (Caprara et al., 2005). The Spanish version used here come from Rodriguez et al. (2017) for the Argentinian population.

### Procedure

#### Data Collection

The study was authorized by the Ethics Committee of the Universitat de València. Participants were contacted at universities in Argentina, Peru, and Spain. If they wished to participate in the research, they were sent a link to an electronic form, where they had to complete a process of informed consent to answer the questionnaires. The entire sample was collected online.

#### Analysis

The analysis was divided into analysis of irrelevant answers, descriptive analysis of item responses, content validity testing on the clarity of the items, scalability of the score and the items, dimensionality of the score, internal consistency of the reliability estimates, and invariance and measurement equivalence.

##### Inattentive/irrelevant responses to content

For the present study, inattentive and irrelevant responses were explored, because answering questionnaires through a web platform has generally been associated with this type of irrelevant response pattern (Johnson, 2005). To identify this problem, the distance *D*^2^ (Mahalanobis, 1936) was used to identify subjects who behaved as multivariate outliers; and to confirm this identification, the variability of intra-individual response was examined (IRV; Dunn et al., 2018). Both are effective techniques for this type of problem (Meade and Craig, 2012) and were implemented using the *careless* program (Yentes and Wilhelm, 2018).

##### Descriptive information

Tests of normality related to symmetry (D’Agostino, 1970) and kurtosis (Bonett and Seier, 2002) were used, as well as descriptive statistics to identify the floor and ceiling of each item.

##### Content validity

This part of the analysis highlighted the clarity of the content. The version of questionnaire used as a baseline of content was validated by Rodriguez et al. (2017). An independent evaluation of the content carried out by the authors indicated that it was phrased without apparent local expressions, and seemed generalizable across the participating groups. However, as (a) Spanish speech is generally characterized by local variations in the use of some words, and (b) there may be discrepancies in assessing clarity between expert judges and the participants themselves (Merino-Soto, 2016), we first corroborated whether the phrasing of the items was clear to the participants. For this purpose, they were given a score clarification form for the items. Each participant read the instructions first, and then scored each item using an ordinal scale of five points, from *Not clear* (1) to *Completely clear* (5). The ratings were analyzed using the V coefficient (Aiken, 1980), and their asymmetric confidence interval was computed using the *ICAiken* program (Merino-Soto and Livia, 2009). This coefficient is often used in content validity studies, to quantify the convergence of qualifying judges between values of 0 (absence of consensus) to 1 (complete consensus). To compare the perceived clarity between the three groups (Argentina, Spain, and Peru), a confidence interval of the difference between the V coefficients was applied (Merino-Soto, 2018). Acceptable clarity was established when the score estimates and the lower limit of the interval were above or equal to 0.60 (Merino-Soto and Livia, 2009).

##### Non-parametric analysis of scalability

To evaluate the fundamental properties of the instrument scores (Brodin, 2014), regardless of the strong presumptions of the latent variable models, a non-parametric approach (Mokken, 1971) was used to analyze the ordinal items of the Prosociality Scale (Molenaar and Sijtsma, 1988). This approach examines the ability of a score to differentiate the ordinal rank of the subjects or items of a measure. Its results are a prerequisite for more demanding parametric approaches (Brodin, 2014; Dima, 2018). There are several useful guides for conducting the analysis with the Mokken approach (e.g., Stochl et al., 2012; Watson et al., 2012; Sijtsma and van der Ark, 2017; Palmgren et al., 2018), but all converge on examining three basic properties for the completion of the *monotonic homogeneity model* (MHM; Sijtsma and van der Ark, 2017): (a) scalability of the items, using the H coefficient (Loevinger, 1948); (b) local independence, in which the responses to the items are not mutually influenced, examined by three conditional association indices, W^(1)^, W^(2)^ and W^(3)^ (Straat et al., 2016); and (c) *monoticity*, that is, the function of incremental relation between the item and the latent attribute, evaluated by comparing the current and expected number of violations of the monotonic model (Mokken, 1971). The adjustment to this model generally uses the CRIT statistic, a diagnostic of the quality of the scale constructed using the weighted sum of several evaluative indicators. The result is a count of violations of the model, which through either a lax (CRIT > 80; van Schuur, 2003) or demanding criterion (CRIT > 40; Molenaar and Sijtsma, 2000), allows the identification of an excess of violations of the model, which would suggest removing the item.

For the selection of items, the following criteria were applied: (1) the point estimate of the coefficient *H* should be at least equal to or greater than 0.40 in the total sample; (2) the point coefficient H should be in at least two countries, equal to or greater than 0.40; (3) the lower limit of the IC in 90%, should be greater than 0.35; (4) no coefficient, in its point estimate or its lower limit, should be less than 0.30. This analytical procedure was performed using the *mokken* program (van der Ark, 2012; R Core Team, 2018).

##### Dimensionality and equivalence/invariance

To strengthen the assessment of dimensionality, the structured equation modeling (SEM) methodology was applied to identify the final characteristics of dimensionality and measurement invariance. To examine the dimensionality, we used a robust estimator for categorical variables (Muthén, 1984), which adjusts the first and second moments of the *χ^2^* statistic (mean-and-variance-adjusted unweighted least squares, or WLSMV; Muthén et al., 1997). This method uses a probit link to define the functional relationship between the items and the construct, as well as polychoric correlations between the items and the thresholds estimation to derive more precise parameters (e.g., factor loading) when the distributional asymmetry is strong (Sass et al., 2014; Li, 2016a,b). Potential changes in the re-specification of the measurement and invariance model were detected by (a) the modification index, at the nominal level 0.05 (WLSMV-*χ^2^* > 3.840), and (b) in statistical power (Saris et al., 2009). IM is also a means of assessing local independence within SEM modeling (Douglas et al., 1998).

The sensitivity of each item with respect to its relation with the construct was estimated by means of a measure equivalent to the signal-to-noise ratio (SNR), which is generally an informative measure of the quality of the item, based on two information components: item discrimination and “noise” (residual variance not relevant to the construct; Ferrando, 2012a,b; Ferrando and Lorenzo-Seva, 2013). The SNR was obtained by squared factor loading (λ*^2^*) on 1-λ*^2^*. This relationship is usually binding with the IRT model (Cheng et al., 2012; Ferrando and Lorenzo-Seva, 2013), and is generally part of the reliability estimation for identifying the maximum variability linked to the construct (Bacon et al., 1995; Hancock and Mueller, 2001).

The heterogeneity of factor loads was tested by adjusting to the tau-equivalent model, implemented with a robust procedure (Yuan and Zhang, 2012) in the *coefficientalpha* program (Zhang and Yuan, 2015). The adjustment of the SEM model was evaluated with several practical indexes and conventional cut points: ≥0.95 for CFI and TLI; ≤0.08 for SRMR (Ullman, 2001). Although RMSEA can be recommended in modeling with categorical variables (Hutchinson and Olmos, 1998), it was not used to decide the adjustment due to its poor performance in models with small degrees of freedom (Kenny et al., 2015; Taasoobshirazi and Wang, 2016).

##### Invariance/measurement equivalence

This procedure was carried out in two phases, which looked at intra-country and inter-country equivalence. The intra-country equivalence was investigated in relation to participant sex, controlling the variability of the attribute effect (measured by the total score); to reduce the effect of cells with a small number of subjects (due to the distribution), the observed conditioning score (total score) was segmented into quintiles. The analysis used was the non-parametric differential item functioning (DIF), implemented with contingency tables for ordinal variables. The partial gamma coefficient was used (*γ^*p*^*; Schnohr et al., 2008), with effect levels defined as weak (>0.15), moderate (0.16–0.30), and strong (>0.31). For the purposes of this study, general interpretation suggestions were used for *γ^*p*^* (e.g., >0.60 = strong, >0.30 = moderate, and ≤0.30 = weak; Healey, 2012). This DIF procedure was required to address the small sample size of the compared groups (Lai et al., 2005; Güller and Penfield, 2009).

After verifying the intra-country equivalence, we continued by analyzing the equivalence between countries, through a sequence of steps appropriate for categorical variables (Wu and Estabrook, 2016), starting with a successive implementation of restrictions on the parameters of the items. The configurational invariance was analyzed first, followed by the cumulative restriction of equal thresholds, then the factorial loads, and finally the residuals. The SEM analyses were carried out with the *lavaan* (Rosseel, 2012) and *semtools* programs (Jorgensen et al., 2018). Since there are still no clear options of fit criteria for index of modification in the comparison of three groups, a liberal criterion was used to reduce the probability of Type I error. In this sense, Rutkowski and Svetina (2013) proposed less restrictive criteria in the comparison of more than two groups (but specifically, ≥ 10): Δ_CFI_, Δ_TLI_ and Δ_RMSEA_, changes less than 0.02; these criteria are similar to those conducted in large-scale studies and comparing more than two groups (OECD, 2014). For comparison purposes, criteria applied to IM were also used between two groups (Chen, 2007): Δ_CFI_ ≤ 0.10 and Δ_TLI_ ≤ 0.10. The convergence of the adjustment indices suggested the decision of indices of modification (IM), but since CFI is optimal in the comparison of nested models (Cheung and Rensvold, 2002) and reduces the Type I error (Elosua, 2011), some doubt can be resolved by the observation of CFI.

##### Reliability

Reliability was estimated at the item level and the score of each subscale. Regarding the items, the attenuated corrected coefficient (Wanous and Reichers, 1996) was used, given its lower bias and computational ease (Zijlmans et al., 2018); the minimum acceptable value is around 0.30 (Zijlmans et al., 2017). At the level of score, coefficients congruent with the non-parametric model were used (MS coefficient; Molenaar and Sijtsma, 1988), along with linear SEM modeling with the coefficient *ω* (Green and Yang, 2009) and bootstrap confidence intervals (500 replications) through the *coefficientalpha* program (Zhang and Yuan, 2015). For comparison purposes, the coefficient α was also calculated.

## Results

### Inattentive/Irrelevant Responses to Content

Applying the Mahalanobis distance measure (*D^2^_*Median*_* = 13.914, min = 1.469, Q3 = 19.898), one participant (Peruvian) was detected with the maximum distance (*D*^2^ = 138.72), and was 1.92 greater than the subject with the shortest distance (*D*^2^ = 72.09). Although the *χ^2^* value was lower than the critical value (gl = 16, Bonferroni-α = 0.05, *n* = 46.03), the individual variability (IRV coefficient) for this participant corresponded with the maximum value of individual deviation (IRV = 1887), and it was also consistent in the identification of *D*^2^. To reduce the probability that the identified participant was a “positive” or “negative” influential case in the adjustment due to its magnitude compared with the rest of the participants (Pek and MacCallum, 2011), this participant was removed, leading to a total sample of 736 for the following analyses.

### Clarity of the Items

Table 1 shows the results of the evaluation of item clarity, as part of the content validity analysis. The point estimate of the coefficients was universally over 0.70, and their asymmetric confidence intervals were predominantly over 0.60; this is a minimally acceptable level (Merino-Soto and Livia, 2009). The average clarity in each group showed similarity between Argentinian and Spanish students (about 0.82), while it was comparatively low in Peruvian students (below 0.80), but nonetheless still at a satisfactory level of perceived clarity. For some items, the lower limit of the IC was below 0.60 (item 5 in Spain, item 11 in Peru, and item 8 in the three groups). These items were reviewed by the authors, especially item 8, where the psychometric behavior was observed in order to determine the effect of this relatively low perceived clarity. In the comparison between groups (through confidence intervals of the difference, in agreement with Merino-Soto, 2018), the most frequent discrepancies occurred among Peruvian students (perceived lower clarity) compared to Spanish and Argentinians, but the point estimates and their intervals in Peruvians tended to be acceptable. The lower limit of the interval for several items was around 0.05, indicating that in the population the difference detected might be small. At this stage, it was concluded that the clarity of the instrument was essentially satisfactory in the three groups.

**TABLE 1:** Coefficients V: clarity of content between participants (Argentina, Spain, and Peru).

	Coefficients V (IC 90%)									Confidence interval for differences in V (90%)					
	Argentina (*n* = 23)			Spain (*n* = 24)			Peru (*n* = 23)			Arg. − Spa.		Arg. − Peru		Spa. − Peru	
	V	L	U	V	L	U	V	L	U	L	U	L	U	L	U
Ps1	0.880	0.813	0.925	0.875	0.809	0.920	0.837	0.765	0.891	–0.076	0.085	–0.043	0.128	–0.047	0.123
Ps2	0.935	0.879	0.966	0.918	0.859	0.953	0.805	0.729	0.864	–0.049	0.084	**0.049**	**0.212**	**0.030**	**0.197**
Ps3	0.935	0.879	0.966	0.938	0.884	0.967	0.857	0.787	0.907	–0.066	0.059	**0.003**	**0.155**	**0.007**	**0.157**
Ps4	0.913	0.852	0.95	0.855	0.786	0.904	0.773	0.693	0.836	–0.020	0.136	**0.052**	**0.228**	–0.011	0.176
Ps5	0.837	0.765	0.891	**0**.**668**	**0**.**585**	**0**.**741**	0.740	0.659	0.808	**0.066**	**0**.**268**	–0.002	0.194	–0.179	0.037
Ps6	0.880	0.813	0.925	0.885	0.821	0.928	0.805	0.729	0.864	–0.085	0.073	–0.014	0.163	–0.007	0.167
Ps7	0.750	0.669	0.816	0.720	0.639	0.789	0.728	0.645	0.797	–0.076	0.134	–0.084	0.128	–0.114	0.100
Ps8	**0**.**620**	**0**.**534**	**0**.**699**	**0**.**520**	**0**.**437**	**0**.**602**	**0**.**663**	**0**.**578**	**0**.**738**	–0.019	0.215	–0.157	0.073	–0.255	–0.025
Ps9	0.958	0.908	0.981	0.845	0.775	0.896	0.837	0.765	0.891	**0.042**	**0**.**187**	**0.047**	**0.197**	–0.080	0.096
Ps10	0.945	0.892	0.973	0.970	0.926	0.988	0.815	0.740	0.872	–0.081	0.027	**0.052**	**0.210**	**0.083**	**0.232**
Ps11	0.880	0.813	0.925	0.813	0.739	0.869	**0**.**675**	**0**.**591**	**0**.**749**	–0.020	0.154	**0.105**	**0.30**	**0.033**	**0.239**
Ps12	0.825	0.751	0.881	0.875	0.809	0.920	0.695	0.611	0.767	–0.137	0.037	**0.027**	**0.231**	**0.082**	**0.275**
Ps13	0.945	0.892	0.973	0.938	0.884	0.967	0.783	0.704	0.845	–0.053	0.068	**0.08**	**0.246**	**0.073**	**0.239**
Ps14	0.837	0.765	0.891	0.698	0.616	0.768	0.750	0.669	0.816	**0.039**	**0**.**237**	–0.011	0.184	–0.157	0.055
Ps15	0.958	0.908	0.981	0.885	0.821	0.928	0.815	0.740	0.872	**0.007**	**0**.**141**	**0.067**	**0.221**	–0.016	0.156
Ps16	0.958	0.908	0.981	0.918	0.859	0.953	0.847	0.776	0.899	–0.021	0.103	**0.039**	**0.186**	–0.008	0.150
Media	0.879	–	–	0.833	–	–	0.777	–	–	–	–	–	–	–	–

*Arg., Argentina; Spa., Spain; bold values, point coefficients below 0.70, lower interval below 0.60, or statistically significant difference; L, lower interval; U, upper interval.*

### Descriptive Statistics of the Items

Table 2 shows the items were distributed asymmetrically, with the highest density in the high response options; in the total sample, the asymmetry coefficients ($\sqrt{b_{1}}$) varied between −0.210 (item 11) and −1.065 (item 10). The kurtosis (*b*_2_−3) showed more variability, with positive and negative values, and between −0.496 (item 11) and 1.041 (item 2). Overall, the items showed moderate or strong departures from normality (D’Agostino-Pearson *K*^2^ between 15.3 and 112.5, *p* < 0.01).

**TABLE 2:** Statistical descriptive information of items.

	Peru						Spain						Argentina					
	*M*	*SD*	Min	Max	Floor	Ceiling	*M*	*SD*	Min	Max	Floor	Ceiling	*M*	*SD*	Min	Max	Floor	Ceiling
Ps1	3.941	0.81	1	5	0.50	22.40	4.255	0.72	2	5	1.00	41.00	3.961	0.86	1	5	0.00	28.50
Ps2	4.114	0.84	1	5	0.50	35.60	4.445	0.65	1	5	0.30	51.90	4.184	0.86	2	5	0.00	42.00
Ps3	4.105	0.80	2	5	0.00	32.40	4.432	0.69	2	5	0.00	53.50	4.280	0.81	2	5	0.00	47.80
Ps4	3.836	1.00	1	5	3.20	27.40	3.735	1.02	1	5	3.20	24.80	3.652	1.17	1	5	4.30	30.00
Ps5	3.804	0.96	1	5	1.80	26.50	4.248	0.80	1	5	0.30	43.90	3.792	1.04	1	5	3.40	27.50
Ps6	3.890	0.83	2	5	0.00	25.10	4.016	0.78	2	5	0.00	28.10	3.792	0.91	1	5	1.00	23.20
Ps7	3.658	0.91	1	5	2.30	16.40	3.600	0.81	1	5	0.60	11.90	3.304	0.96	1	5	3.90	10.10
Ps8	3.845	0.86	1	5	0.90	23.70	4.435	0.77	1	5	0.30	57.40	4.039	0.91	1	5	1.00	36.20
Ps9	3.982	0.75	2	5	0.00	24.20	4.226	0.69	2	5	0.00	36.50	4.179	0.87	1	5	0.50	44.00
Ps10	3.945	0.94	1	5	0.50	30.10	4.455	0.65	2	5	0.00	53.20	4.256	0.85	1	5	1.00	46.90
Ps11	3.233	1.05	1	5	4.10	11.00	3.448	0.93	1	5	1.90	12.30	3.338	1.11	1	5	5.80	16.90
Ps12	3.594	0.96	1	5	2.30	16.00	4.077	1.01	1	5	4.50	37.40	3.705	1.06	1	5	3.40	27.10
Ps13	3.877	0.82	2	5	0.00	23.70	4.165	0.74	1	5	0.60	34.20	3.932	0.87	1	5	0.00	27.50
Ps14	4.046	0.77	2	5	0.00	27.40	4.335	0.65	3	5	0.00	43.20	4.164	0.89	1	5	0.50	42.50
Ps15	4.000	0.81	1	5	0.50	28.60	4.410	0.69	2	5	0.00	51.00	4.116	0.87	1	5	1.00	37.70
Ps16	4.009	0.91	1	5	1.40	32.90	4.342	0.69	2	5	0.00	45.50	4.203	0.87	1	5	0.50	44.40

*M, mean; SD, standard deviation; Min, minimum score; Max, maximal score.*

In relation to some demographic variables (sex and age), in the total sample the Spearman correlation between the items and age was around zero (between −0.06 and 0.064), and predominantly without statistical significance. In each group, this trend was similar (Argentina: median = 0.042; Peru: median = −0.039; Spain: median = −0.024). Regarding sex, Spearman correlations varied between 0.030 (item 9) and 0.189 (item 4, female > male), and in each country it was also predominantly close to zero in Peru (median = 0.032), but around 0.10 in Argentina (median = 0.118) and Spain (median = 0.159). Finally, due to the tendency of responses toward high scores, several items in each country showed a ceiling effect, such that the minimum response was frequently option 2 or 3, especially in Spain and Peru. To align the analysis of latent variables with the methodology for categorical variables, options 1 and 2 were therefore integrated on these items, leaving the rest unmodified.

### Non-parametric Analysis

#### Scalability

Regarding scalability (Table 3), in the first iteration of the analysis several items showed *H* scores below 0.40 in the three countries, as well as low levels of scalability in their confidence intervals (items 2, 9, 11, 12, and 16); other items showed comparatively weak *H* in at least two countries (items 1, 4, and 14). These items thematically corresponded to behaviors of sharing personal resources (2, 9, 11, and 14), taking another’s perspective in situations of discomfort (i.e., empathy; 12 and 16), and comfort and willingness to give help to others (1 and 4). The items that were satisfactorily maintained according to the initial criteria were items 3, 5, 6, 7, 8, 13, and 15, whose contents were distributed over helping behaviors (3, 6, and 7), empathy (5 and 8), and giving supportive company to others (13 and 15). Although item 10 (interpreted as providing help through emotional comfort) partially met the initial criteria, it was not included in the resulting version so as not to overemphasize the “helping” component in the instrument score. In the left section of Table 3, the results of the final iteration are shown. The scalability coefficient for the scale was 0.50 in the countries, and around 0.50 for each item (except item 15 that tended to be a little lower, though still close to 0.50). All were statistically significant with an alpha of 0.05 (for the items, *z* between 28.88 and 33.83; for the total score, *z* = 59.83).

**TABLE 3:** Results of Mokken non-parametric analysis (scalability and monoticity).

	Scalability								Scalability								Monoticity		
	(*H* coefficient, first iteration)								(*H* coefficient, second iteration)								(*n* = 736)		
	Total sample		Argentina		Spain		Peru		Total sample		Argentina		Spain		Peru		#vi	#z_sig_	CRIT
	(*N* = 736)		(*N* = 207)		(*N* = 310)		(*N* = 219)		(*N* = 736)		(*N* = 207)		(*N* = 310)		(*N* = 219)				
	*H*	*se*	*H*	*se*	*H*	*se*	*H*	*se*	*H*	*se*	*H*	*se*	*H*	*se*	*H*	*se*			
Ps1	0.402	0.025	0.374	0.049	0.345	0.032	0.432	0.046											
Ps2	0.379	0.026	0.333	0.050	0.275	0.032	0.465	0.041											
Ps3	0.492	0.022	0.503	0.041	0.408	0.030	0.526	0.038	0.571	0.025	0.590	0.045	0.493	0.039	0.592	0.041	0	0	0
Ps4	0.361	0.025	0.400	0.042	0.295	0.033	0.40	0.046											
Ps5	0.432	0.026	0.407	0.053	0.393	0.033	0.430	0.047	0.524	0.028	0.506	0.058	0.520	0.039	0.484	0.050	0	0	0
Ps6	0.455	0.024	0.467	0.044	0.410	0.031	0.460	0.044	0.560	0.025	0.563	0.046	0.545	0.039	0.542	0.043	0	0	0
Ps7	0.458	0.024	0.443	0.046	0.397	0.029	0.532	0.040	0.557	0.027	0.560	0.049	0.513	0.038	0.588	0.047	0	0	0
Ps8	0.467	0.023	0.454	0.045	0.386	0.033	0.487	0.042	0.555	0.025	0.547	0.051	0.523	0.037	0.527	0.047	0	0	0
Ps9	0.388	0.026	0.368	0.050	0.283	0.027	0.486	0.043											
Ps10	0.443	0.025	0.414	0.052	0.368	0.031	0.472	0.041											
Ps11	0.333	0.024	0.349	0.042	0.260	0.037	0.363	0.044											
Ps12	0.343	0.029	0.398	0.047	0.180	0.048	0.405	0.047											
Ps13	0.498	0.021	0.509	0.038	0.447	0.031	0.496	0.037	0.575	0.023	0.577	0.043	0.585	0.035	0.520	0.04	0	0	0
Ps14	0.399	0.025	0.371	0.045	0.306	0.031	0.475	0.043											
Ps15	0.45	0.023	0.458	0.038	0.342	0.032	0.487	0.044	0.487	0.028	0.480	0.052	0.397	0.045	0.520	0.04	0	0	0
Ps16	0.348	0.028	0.293	0.052	0.260	0.033	0.432	0.047											
H	0.413	0.019	0.407	0.036	0.33	0.023	0.456	0.035	0.546	0.022	0.545	0.043	0.512	0.031	0.537	0.040			

*se, *H* standard error; #vi, number of violations to monoticity; #z_*sig*_, number of statistically significant violations; CRIT, combined count of #vi y #z_*sig*_.*

#### Local Independence

In the analysis of conditional association (not shown in Table 3), the indices *W^(2)^* and *W^(3)^* did not detect any violation of local independence. Violations were found for *W^(1)^* between item 8 and items 5 (*W^(1)^* = 12.191), 9 (*W^(1)^* = 10.227), 12 (*W^(1)^* = 12.485) and 16 (*W^(1)^* = 10.124), and between item 13 and items 12 (*W^(1)^* = 13.096) and 16 (*W^(1)^* = 13.349). To corroborate this, within the next dimensionality analysis the indices of modification were evaluated.

#### Monotony

Finally, no violation of monotony was detected in the version obtained from seven items (see left side of Table 3). Based on the results of the non-parametric analysis as a whole, the obtained version had the following characteristics: the scalability of the score in the total sample and in each country was greater than 0.50, and its population variability was greater than 0.48, while each item showed a moderately similar magnitude of scalability, but generally greater than 0.50.

### Dimensionality and Equivalence/Invariance

#### Analysis of Dimensionality (SEM)

Because the Prosociality Scale was apparently designed as a congeneric one-dimensional measure (without restriction of statistical equality between its items), the evaluation of the adjustment started with this model. The adjustment of the congeneric model with the 16 complete items was satisfactory according to the practical indices measure (see Table 4, results of the full version). The analysis of the modification indices indicated that potential mis-specifications were inconsistent according to the criteria of statistical power and practical significance (Saris et al., 2009). Given the strength of the adjustment and some trivial mis-specifications, this model was initially retained without add re-specifications. Although all the factorial loadings were statistically significant (*z* > 10.0), they varied from 0.500 to 0.811, which related to a large amount of variance in the construct (between 0.250 and 0.658, respectively). This suggested a wide range of variability (levels of 0.40, 0.50, 0.60, and 0.80; Beauducel and Wittmann, 2005). The SNR for each item emphasized the difference between the factorial loads, varying from 0.333 to 1.992, suggesting that the information relevant to the represented construct could range between very weak and very strong.

**TABLE 4:** Dimensionality (CFA-SEM) and differential item functioning (DIF).

	Dimensionality (CFA – SEM)						Differential item functioning (DIF)						Item-score reliability			
	Full version			Short version			Peru		Spain		Argentina		Total	Peru	Spain	Argentina
	(*n* = 736)			(*n* = 736)			(*n* = 219)		(*n* = 310)		(*n* = 207)					
	λ	*h*^2^	SNR	λ	*h*^2^	SNR	*γ^*p*^*	H-*χ^2^*	*γ^*p*^*	H-*χ^2^*	*γ^*p*^*	H-*χ^2^*				
Ps1	0.653	0.426	0.743													
Ps2	0.627	0.393	0.648													
Ps3	0.800	0.640	1.778	0.776	0.601	1.514	0.374**	4.47	0.231	1.87	0.241	1.63	0.527	0.548	0.437	0.579
Ps4	0.567	0.321	0.474													
Ps5	0.734	0.538	1.168	0.777	0.603	1.524	0.175	5.04	0.441**	10.66	–0.184	14.93	0.458	0.404	0.475	0.418
Ps6	0.715	0.511	1.046	0.757	0.573	1.342	0.051	13.26	–0.166	5.81	–0.225	13.47	0.499	0.548	0.453	0.513
Ps7	0.701	0.492	0.966	0.722	0.522	1.089	–0.175	8.07	–0.098	1.62	–0.355	1.36	0.462	0.556	0.427	0.521
Ps8	0.811	0.658	1.922	0.824	0.678	2.115	–0.217	4.91	0.152	4.12	0.378	8.68	0.527	0.516	0.490	0.513
Ps9	0.615	0.378	0.608													
Ps10	0.718	0.515	1.064													
Ps11	0.500	0.250	0.333													
Ps12	0.591	0.349	0.537													
Ps13	0.797	0.635	1.741	0.799	0.639	1.765	0.341	10.15	–0.040	1.48	0.233	2.49	0.552	0.474	0.577	0.569
Ps14	0.666	0.444	0.797													
Ps15	0.733	0.537	1.161	0.671	0.450	0.819	–0.004	0.38	0.163	2.45	0.097	1.51	0.379	0.452	0.255	0.370
Ps16	0.566	0.321	0.471													
*χ^2^*	646.382			150.672												
(gl)	(104)			(14)												
CFI	0.980			0.985												
TLI	0.977			0.977												
RMSEA	0.084			0.115												
SRMR	0.065			0.063												

λ, factor loading; *h*^2^, total variance; SNR, signal-to-noise ratio; *χ^2^*, WLSMV stimator; H-*χ^2^*, strata homogeneity test of quintile score; *γ^*p*^*, gamma partial coefficient. **p < 0.007.

According to the results of the non-parametric analysis, a second iteration of the confirmatory factor analysis (CFA) was conducted, and the results of the model adjustment are shown in Table 4 (results of the reduced version). These indicate a satisfactory adjustment, which was practically similar in the specific indices compared with the full version (Δ_CFI_ = 0.005, Δ_TLI_ = 0.001, Δ_SRMR_ = 0.004). The adjustment without the recategorized items was also satisfactory, WLSMV-*χ^2^* = 155.3 (gl = 14, *p* < 0.01; CFI = 0.985, TLI = 0.978, SRMR = 0.061). These results were superior to the adjustment criteria chosen. All factorial loads were greater than 0.60, varying between 0.675 and 0.822; the change of the loads compared with the loads of the full version varied between | 0.1%| and | 7.9%|, while the factor loading of items 5, 6, 7, and 8 showed a small increase (between 1.4 and 5.7%). The adjustment with the reclassified items was indistinguishable from the results obtained before recategorization of the items (see Table 4).

After the congeneric modeling, in the adjustment of the tau-equivalent model, the common factor load were estimated as 0.764 (*h*^2^ = 0.583). The adjustment was WLSMV-*χ^2^* = 207.8 (gl = 20, *p* < 0.01), CFI = 0.980, TLI = 0.979, SRMR = 0.069, RMSEA = 0.113 (IC 90% = 0.099, 0.127). Although the statistical test of tau-equivalence (Yuan and Zhang, 2012) rejected the null hypothesis of accepting this model, the differences of this model versus the congeneric model can be considered trivial: Δ_CFI_ = 0.005, Δ_TLI_ = 0.001, Δ_SRMR_ = 0.008.

#### Equivalence and Measurement Invariance

The intra-country analysis (see left part of Table 4) found that, once we controlled the performance on the observed score for the number of statistical tests (Bonferroni adjustment, *p* = 0.007), the tendency of the partial gamma coefficients (*γ^*p*^*) was essentially concentrated on the weak level (≤0.30). The items detected by possible uniform DIF (3 in Peru, and 5 in Spain) were examined in their content, and it was established that there was no reason to recognize any potential sources of DIF; therefore at this stage they were dismissed. On the other hand, although there were variations in the magnitude of the *γ* coefficient (not shown here) across quintiles, the homogeneity of the coefficients in the quintiles was confirmed (H-*χ^2^* < 15.0, Bonferroni adjusted *p* = 0.007), suggesting absence of non-uniform DIF.

Regarding the invariance/equivalence between countries, the baseline (configurational) model, along with the remaining models that included cumulative constraints, showed that the compared parameters (factorial loads, thresholds and residuals) changed only trivially (Table 5). Considering the chosen criteria (Chen, 2007; Rutkowski and Svetina, 2013; OECD, 2014), the equality constraints for each level of invariance produced results that suggested no invariance, and therefore it was concluded that there was compliance with the invariance across the three levels evaluated.

**TABLE 5:** Results of between invariance/equivalence (countries).

Invariance steps	WLSMV-*χ^2^* (gl)	CFI	TLI	SRMR	Δ_CFI_	Δ_TLI_	Δ_SRMR_
Configurational	186.421 (42)	0.985	0.977	0.075	–0.009	–0.005	0.012
Weak (Metric)	284.553 (54)	0.976	0.972	0.087	0.000	0.009	–0.01
Strong (Scalar)	311.929 (80)	0.976	0.981	0.077	–0.008	–0.003	0.010
Strict	404.714 (94)	0.968	0.978	0.087	–0.009	–0.005	0.012

*Δ, differences between fit indices CFI, TLI, and SRMR.*

### Reliability

In the total sample, we obtained an *ω* of 0.865 (*SE* = 0.009; 95% CI = 0.844,0.880); while α was 0.864 (SE = 0.009; 95% CI = 0.847,0.880). For practical purposes the two were indistinguishable. Estimated for each country, in Argentina (*ω* = 0.870, *SE* = 0.018, 95% CI = 0.830,0.899), Peru (*ω* = 0.890, *SE* = 0.016, 95% CI = 0.831,0.894), and Spain (*ω* = 0.845, *SE* = 0.015, 95% CI = 0.811,0.869), the coefficients were very similar and the variation could be due to sampling error. The α coefficients for each country (respectively 0.869, 0.842, and 0.869) showed insubstantial differences with the estimates of *ω*. The item-level reliability showed consistently high results in Argentina (median = 0.513, min. = 0.370, max. = 0.578), Peru (median = 0.516, min. = 0.403, max. = 0.556) and Spain (median = 0.452, min. = 0.255, max. = 0.577), and was similar between all three countries. Across the sample as a whole, the results were acceptable (see lower left side of Table 4).

## Discussion

The present study applied psychometric methodology and rational-theoretical evaluations to refine the Prosociality Scale constructed by Caprara et al. (2005) for adult populations. Given the cross-cultural context of this study, it was particularly challenging to show the invariance of the scale’s psychometric properties, and to date this is the only attempt at a cross-cultural psychometric exploration of the scale across several Spanish-speaking countries.

When the items were examined, they were characterized as not being distributed normally, characteristically with negative asymmetry. Also, the answers were oriented toward high response options. This trend was similar among the three countries examined. Associations with age were predominantly distributed around zero, both in the total sample and within individual countries. In contrast, relationships with sex were predominantly small in Spain (women > men), between trivial and small in Argentina (women > men), and completely trivial (around zero) in Peru. Considering that the differences in functioning of the items were trivial with respect to the sex of the participants, this finding for some individual items could lead to future explorations of differences at the level of the total score, but due to the strong asymmetry in the sex distribution in our samples, it would be best to avoid overinterpreting these results.

The fundamental psychometric criteria of our study were first based on a non-parametric method, created to evaluate the properties of measures that serve for ordering people based on their observed scores. Interestingly, the results of the application of the SEM and Mokken methodologies showed two things: first, they tended to show convergence in the items with lower scalability and covariation with the construct, as identified in the study by Caprara et al. (2005); and second, items with comparatively poorer properties were more clearly identified by the non-parametric method (Mokken). Specifically, with the SEM method the items in general showed factor loads that are usually acceptable in the literature (>0.30 or >0.40), while these same levels applied to the *H* coefficient suggested a low scalability, and therefore lessened the discriminative ability of the observed score.

The content of the resulting scale was distributed over behaviors subsumed along one dimension, partially converging with the logic of another prosociality instrument created in one of the participating countries (Argentina), which is also applicable to university students (Auné et al., 2014). In that study, the instrument was multidimensional, with correlations between weak and moderate in the heterogeneous item-construct relationship (factorial loads). The two dimensions identified were interpreted as representing empathic behavior on the one hand, and initiative to help people on the other. In its analytical exploration, the former eigenvalue was very large in relation to the remaining values, and could suggest the exploration of a general latent variable, or that items with common variance load strongly toward a latent general factor. However, the difference between the one-dimensional model proposed here, and the multidimensional model proposed in the study of Auné et al. (2014) is influenced by the design of the theoretical constructions, and a combination of *post hoc* conceptual and empirical criteria to refine each instrument. Nevertheless, in our opinion the higher-order construct is prosocial behavior, supported by strongly intercorrelated specific content items. Thus, in the present study, conceptual decisions balanced purely empirical and mathematical decisions.

One of the evaluated characteristics was the adjustment to a tau-equivalent model (constraint of equality of factorial loads) compared with a congeneric model (in which factor loads were free to vary), which allowed us to identify the similarity in the construct representation of items and the appropriate reliability models. As in other Latin American studies (e.g., Auné et al., 2014, 2016), the heterogeneity of factor loads led to doubt about the appropriateness of internal consistency estimates such as the alpha coefficient, which assume the tau-equivalent model among the items. In the present study, although the statistical test of the difference between the congeneric and tau-equivalent models was statistically significant, the practical discrepancies between the two did not seem to be moderate or strong, but rather trivial. This leads to the conclusion that the items essentially showed similarity in their representativeness of the construct, and similar sensitivity to differentiate individual variability in the measured attributes. An additional advantage of adjusting the scale to a tau-equivalent model is that it helped to recover weak factorial models (Ximénez, 2006, 2016), and to avoid the rejection of models with salient factorial loading of 0.50 or less (Beauducel and Wittmann, 2005). Therefore, it is possible that the structure of the present version of the instrument can be replicated in future studies.

There are discrepancies in results regarding differences in prosociality according to participant sex (Martí-Vilar and Lorente, 2010). Some authors have argued that women show higher levels of prosociality, differences that are more marked in adult life (e.g., Eisenberg and Fabes, 1998). Other authors have noted that these sex differences depend on the motivation or type of prosocial behavior (Carlo et al., 2003; Auné et al., 2017). A plausible hypothesis that could explain this inconsistency is that certain instrument items but not others are psychometrically invariant. However, this was not verified in previous studies.

Although this study was carried out on a Spanish-speaking population, there are many differences between the societies of Spain, Argentina and Peru. Carballeira et al. (2014) showed that Latin American societies are more influenced by a collectivist culture, while Spanish society is more influenced by individualism. Such differences allow us to see the importance of this study since it involved testing the Prosociality Scale in countries with diverse cultural characteristics.

Due to the inconsistency of findings on the effect of sex on the variability of self-reported prosocial behavior, the investigation of equivalence was a preliminary, *sine qua non*, stage for the new version of the instrument. We found that, once the effect of the total score (measured as such) was controlled (using the DIF analysis approach), the differences in the answers were not outside the level of sampling error, and were generally trivial in magnitude. In the Peruvian and Spanish participants, two items worked differentially when the effect of the total score was controlled. Although the statistical detection of DIF does not directly indicate the absence of real bias (Lai et al., 2005), this is an avenue for further investigation. A qualitative analysis was beyond the objectives of this study, and thus the sources of this differential functioning were not qualitatively explored, so the conclusion of equivalence between men and women within each country is something to be tested by subsequent studies. Although this conclusion should be interpreted in the context of the limitations of the study (sample size and asymmetric proportion of men and women in each country), our results with the new reduced version can also be considered internally valid due to the strength of the unidimensional measurement model. As previously found, the unidimensionality of the new version is characterized by items with strong factorial loads, high signal-to-noise ratio, and an interdependent content relating to different observed behaviors.

Regarding the limitations of the study, one of these is the sample size. This can be considered large (>500) in terms of the total group size (Finch and French, 2008; Ximénez, 2016; Finch et al., 2018), but for the intra-country analysis it can be considered small (Ximénez, 2016). This could explain certain idiosyncratic variations between countries found in this sample. The intra-country sample sizes of our study, however, are typical of the common situation of small (or moderate) samples in social science research, and particularly in psychology (Beauducel and Wittmann, 2005). As more generally in psychology, the sample size of this study, in the total sample and in each subgroup, may generate suboptimal conditions for estimating psychometric parameters and their potential replicability. Although this problem is shared with many studies in the social sciences in general, and in psychology in particular (Beauducel and Wittmann, 2005), other aspects should also be considered to evaluate the potential replicability of our results: for example, the high magnitude of the factorial loading, as well as the convergence between the methodologies that were applied, and between the levels of statistical significance and practical significance that were found. Indeed, the application of several methods to identify dimensionality (within a framework of sensitivity analysis) can lead to more confidence in the results obtained, given the convergence observed.

A second limitation of the study is the asymmetric proportionality between men and women. However, the distribution of men and women in the sample may reflect current sex distributions among undergraduate students of psychology in Argentina, Spain, and Peru (and indeed other countries). Anecdotal evidence from the authors regarding said distribution supports this idea. A third limitation was the criterion used to decide on measurement invariance, since although the results of the adjustment met conventional criteria (≥0.90 or 0.95, Hu and Bentler, 1999) and other revised criteria (≥0.96; Yu, 2002), such criteria continue to be the subject of debate and further methodological research. This seems to be more prominent when comparing more than two groups (but less than ten), and in the context of asymmetric distribution of participants and moderately small sample size. The criteria applied in the present study (Chen, 2007; Rutkowski and Svetina, 2013; OECD, 2014) might produce Type I or II errors, and our criterion was essentially liberal. As the present study is one of the first of its kind, this decision should be re-evaluated for future studies. However, one aspect that balances this problem was that the approach of evaluating invariance/equivalence (applied to categorical variables) tends to yield robust and sensitive performance (Kim and Yoon, 2011; Sass et al., 2014). Another limitation is that the possible effect of the social desirability of the responses was not verified; this problem may have been reduced by the anonymity of data collection, or it may show correlations between moderate or weak (Rodrigues et al., 2017), and the reader is suggested to assess our results in the context of this limitation. Finally, other evidences of validity are required to corroborate the theoretical representation of this modified version of the instrument. Future studies should focus on the limitations of the study to advance the replicability of the results, as well as to obtain other evidence of validity required to open the way to substantive research with the instrument re-constructed here. This would contribute to our knowledge of prosociality measures, which are still an emerging area of investigation in measurement issues (Martí-Vilar et al., 2019).

## Data Availability Statement

The datasets generated for this study are available on request to the corresponding author.

## Ethics Statement

This study was carried out in accordance with the recommendations of the United Nations Educational, Scientific and Cultural Organization (UNESCO), Declaration of Helsinki, and indicators of the Ethics Committee of the Universitat de València, No. H14820253925 (February 2, 2017). The studies involving human participants were reviewed and approved by the Ethic Committee of Universitat de Valéncia. The patients/participants provided their written informed consent to participate in this study.

## Author Contributions

MM-V, CM-S, and LR designed the research and collected the data. CM-S analyzed the data. CM-S and LR interpreted the data. MM-V, CM-S, and LR drafted the manuscript. All authors critically revised the manuscript and gave their approval to the final version to be published.

## Conflict of Interest

The authors declare that the research was conducted in the absence of any commercial or financial relationships that could be construed as a potential conflict of interest.
